# Supplementary material for: Inactivation of SARS-CoV-2 and COVID-19 Patient Samples for Contemporary Immunology and Metabolomics Studies
Source: Immunohorizons. Author manuscript; Available in PMC 2022 Jun 3. (PMC9164212; doi:10.4049/immunohorizons.2200005)
Supplement: 1 [file NIHMS1811821-supplement-1.pdf]

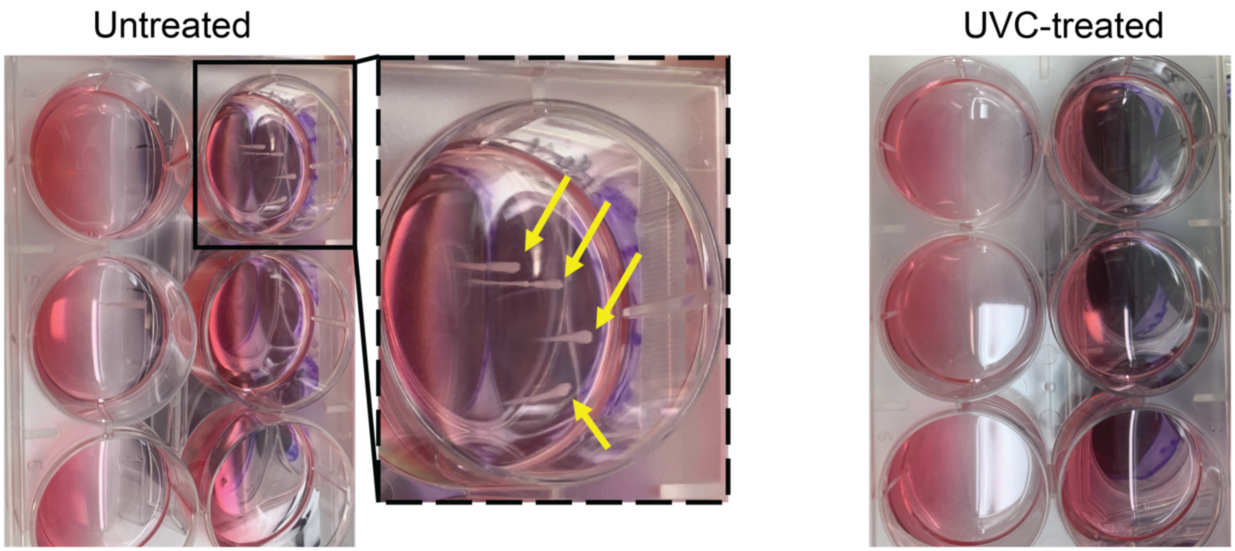

Figure S1. ***UVC inactivation abolishes microbial growth in plaque assay cultures*.** Representative image of plaque assay cultures for respiratory supernatant samples (severe COVID-19 patients) before and after UVC-treatment (30 min at ~4000 µwatt/cm^2). Yellow arrows indicate microbial growth.

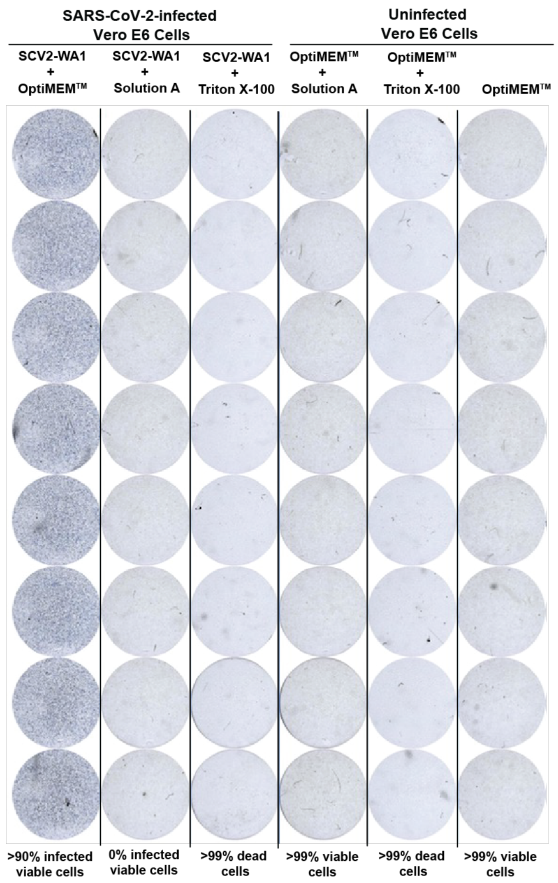

Figure S2. *Metabolite extraction solvent (Solution A) at a decreased concentration completely inactivates SARS-CoV-2*.
FRNA results evaluating SARS-CoV-2 inactivation using 2 volumes (versus 4 volumes shown in Fig. 3) of the metabolite extraction solvent (Solution A) to 1 volume of stock virus (7.5 x 10^6 $TCID_{50}$/mL) in the standard metabolomic sample processing procedure (see methods) and Triton X-100.
